# Pharmacological Discrimination of Effects of MK801 on Thalamocortical, Mesothalamic, and Mesocortical Transmissions

**DOI:** 10.3390/biom9110746

**Published:** 2019-11-18

**Authors:** Motohiro Okada, Kouji Fukuyama, Tomosuke Nakano, Yuto Ueda

**Affiliations:** Department of Neuropsychiatry, Division of Neuroscience, Graduate School of Medicine, Mie University, Tsu 514-8507, Japan; k-fukuyama@clin.medic.mie-u.ac.jp (K.F.); t-nakano@clin.medic.mie-u.ac.jp (T.N.); uedayuto@gmail.com (Y.U.)

**Keywords:** *N*-methyl-d-aspartate, schizophrenia, mood disorder, l-glutamate, GABA, monoamine

## Abstract

*N*-methyl-d-aspartate/glutamate receptor (NMDAR) is one of the major voltage-sensitive ligand-gated cation channel. Several noncompetitive NMDAR antagonists contribute to pathophysiology of schizophrenia and mood disorders; however, the effects of inhibition of NMDAR on several transmitter system have not been well clarified. Thus, this study determined the selective NMDAR antagonist, MK801 (dizocilpine), on thalamocortical, mesothalamic, and mesocortical transmissions associated with l-glutamate, GABA, serotonin, norepinephrine, and dopamine using multiprobe microdialysis. Perfusion with MK801 into the medial prefrontal cortex (mPFC) increased and decreased respective regional releases of monoamine and GABA without affecting l-glutamate. The mPFC MK801-induced monoamine release is generated by the regional GABAergic disinhibition. Perfusion with MK801 into the reticular thalamic nucleus (RTN) decreased GABA release in the mediodorsal thalamic nucleus (MDTN) but increased releases of l-glutamate and catecholamine without affecting serotonin in the mPFC. The RTN MK801-induced l-glutamate release in the mPFC was generated by GABAergic disinhibition in the MDTN, but RTN MK801-induced catecholamine release in the mPFC was generated by activation of α-amino-3-hydroxy-5-methyl-4-isoxazolepropionate/glutamate receptor (AMPAR) which received l-glutamate release from thalamocortical glutamatergic terminals in the mPFC. Perfusion with MK801 into the dorsal raphe nucleus (DRN) decreased GABA release in the DRN but selectively increased serotonin release in the MDTN and mPFC. These DRN MK801-induced serotonin releases in the both mPFC and MDTN were also generated by GABAergic disinhibition in the DRN. These results indicate that the GABAergic disinhibition induced by NMDAR inhibition plays important roles in the MK801-induced releases of l-glutamate and monoamine in thalamic nuclei and cortex.

## 1. Introduction

Recently, the importance of *N*-methyl-d-aspartate/glutamate receptor (NMDAR) modulation has been a source of scientific discussion in psychiatry and psychopharmacology, since various clinical studies have demonstrated that a noncompetitive NMDAR antagonist, ketamine contributes to pathophysiology of schizophrenia and mood disorders [1,2,3,4,5,6,7,8,9]. Glutamatergic transmission abnormalities associated with NMDAR are implicated in the pathophysiology of schizophrenia, as evidenced by induction of schizophrenia-like positive, negative symptoms and cognitive impairments in healthy volunteers, experimental animal models and exacerbation of psychosis in schizophrenia patients by noncompetitive NMDAR antagonists such as phencyclidine and ketamine [1,2,3,4,5,6]. Contrary, (*S*)-ketamine was recently approved by the Food and Drug Administration in early 2019 as a rapid-acting antidepressant against the treatment of major depressive disorders [10]. Furthermore, ketamine has also been shown to have distinct and independent anti-suicidal effects in patients with mood disorders [11,12].

Various targets have been reported for these paradoxical clinical actions of ketamine, although inhibition of NMDAR is considered the main pharmacological target [13,14]. Inhibition of NMDAR activates glutamatergic transmission by GABAergic disinhibition in the cortex and sub-cortical regions [6,7,15,16,17,18,19,20,21]. In our hands, MK801 (selective and potent NMDAR antagonist), also known as dizocilpine, drastically increased serotonin release by also GABAergic disinhibition in the dorsal raphe nucleus (DRN) [16]. Conversely, the 5-HT7 receptor (5-HT7R) antagonists, lurasidone, and SB269970, induced GABAergic disinhibition, but did not increase serotonin release in the presence of 5-HT1A receptor (5-HT1AR) function. Following inhibition of 5-HT1AR in the DRN, both SB269970 and lurasidone increased serotonin release in the DRN, mediodorsal thalamic nucleus (MDTN) and medial prefrontal cortex (mPFC) [15,16]. Although both MK801 and 5-HT7R antagonists exhibit rapid-onset antidepressant-like effects, their actions on serotonin release differ [22]. These differences between the effects of MK801 and lurasidone on serotonergic transmission suggest that GABAergic disinhibition is a candidate major mechanism for rapid-acting antidepressant-like actions rather than enhancement of serotonergic transmission. In spite of these efforts, the detailed mechanisms of double-edge sword clinical action of ketamine regarding the schizophrenia and mood disorder have remained to be clarified.

Traditionally, in spite of inhibition of NMDAR, systemic administration of phencyclidine, ketamine and MK801 drastically increased cortical glutamate release, whereas local administration of these agents into the mPFC or other cortical regions, such as the insular (IsC) or orbitofrontal cortex (OFC), could not affect regional l-glutamate release [15,16,17,18,19,20,21,23,24,25,26]. These contradictive demonstrations suggest that the responsible pathway regarding the l-glutamate release induced by systemic administration of NMDAR antagonists is probably outside of cortical regions; however, the detailed mechanisms of discrepancies the effects between systemic and local administration of NMDAR antagonist on cortical l-glutamate rgic transmission have also remained to be clarified [15,16,17,18,19,20,21,23,24,25,26].

Recently, we have demonstrated the thalamocortical glutamatergic pathway is one of the candidate responsible neural pathways for l-glutamate release in the frontal cortex induced by systemic MK801 administration using multiprobe microdialysis system [15,16,17,18,19,20,21]. One of the major thalamocortical glutamatergic pathway [27] composed of projection form the MDTN to mPFC, IsC, or OFC [17,18,21,23,24,28]. Local administration of 50 μM MK801 into the MDTN increased l-glutamate release in the mPFC, but 10 μM MK801 did not affect [17,21]. MDTN receives GABAergic inhibition from the reticular thalamic nucleus (RTN) [15,18,20,28]. Indeed, local administration of lower than the threshold concentration of MK801 into the RTN decreased the GABA release in the MDTN, but drastically increased l-glutamate release in the mPFC.

The interaction between glutamatergic and monoaminergic transmissions in frontal cortex plays important roles in the functions of neuro cognition and emotional perception in the pathophysiology of schizophrenia and mood disorders [15,16,18,20]. The major and specific noradrenergic, dopaminergic and serotonergic terminals from respective the locus coeruleus (LC), ventral tegmental area (VTA) and DRN project to deeper layers of frontal cortex [23,24]. These specific monoaminergic terminals receive GABAergic inhibition, but not contact with thalamocortical glutamatergic pathway directly [23,24]. Contrary to specific monoaminergic terminals, a part of catecholaminergic terminals (the co-releasing of norepinephrine and dopamine) from the LC projects to superficial layers of frontal cortex [15,23,24]. The co-releasing terminals receive excitatory glutamatergic regulation from thalamocortical glutamatergic pathway. Taken together with these previous findings, we hypothesize that the sensitivities of several transmission systems to NMDAR contributes to the double-edge sword clinical action of NMDAR antagonist against pathophysiology of schizophrenia and mood disorders. Therefore, to clarify the double-edge sword clinical action of NMDAR antagonist on pathophysiology between schizophrenia and mood disorders, the present study determined the threshold concentration of local administration of MK801 on thalamocortical (RTN-MDTN-mPFC) glutamatergic, mesothalamic (DRN-MDTN), and mesocortical (DRN-mPFC) serotonergic transmission associated with NMDAR using multiprobe microdialysis system. Moreover, the regulation mechanisms of these three pathways associated with NMDAR, the effects of α-amino-3-hydroxy-5-methyl-4-isoxazolepropionate/glutamate receptor (AMPAR) and GABA_A_ receptor (GABA_A_-R) on transmitter releases induced by MK801 were also determined.

## 2. Materials and Methods

### 2.1. Preparation of the Microdialysis System

All animal care and experimental procedures were performed in compliance with the ethical guidelines established by the Institutional Animal Care and Use Committee at Mie University (No. 24-35-R3). All studies involving animals are reported in accordance with the relevant ARRIVE guidelines [29]. A total of 120 rats were used in experiments.

Male Sprague-Dawley rats (approximately 250 g, 7−8 weeks old, SLC, Shizuoka, Japan) were maintained in a controlled environment (22 ± 1 °C) with a 12-h light/12-h dark cycle. All rats were weighed before the study. Rats were anesthetized with 1.8% isoflurane and were then placed in a stereotaxic frame for implantation of dialysis probes [30,31]. Concentric direct insertion type dialysis probes (0.22 mm diameter; Eicom, Kyoto, Japan) were implanted in the mPFC (3 mm exposed membrane; A = +3.2 mm, L = +0.8 mm, V = −5.2 mm, relative to the bregma) which comprise infralimbic and prelimbic mPFC, the MDTN (2 mm exposed membrane; A = −3.0 mm, L = +0.9 mm, V = −6.2 mm, relative to the bregma at a lateral angle of 30°), the RTN (A = −1.4 mm, L = +1.2 mm, V = −7.2 mm, relative to the bregma) and the DRN (1 mm exposed membrane; A = −8.2 mm, L = 0.2 mm, V = −6.8 mm, relative to the bregma at a lateral angle of 15°) [15,16]. During recovery and experimentation, rats were housed individually in cages and were provided food and water *ad libitum*. Perfusion experiments were initiated at 18-h after recovery from isoflurane anesthesia [32,33,34]. During experiments, single rats were placed in an in vivo dialysis system for freely moving animals (Eicom) equipped with a two-channel swivel (TCS2-23; ALS, Tokyo, Japan). The perfusion rate was set at 2 μL/min in all experiments using modified Ringer’s solution (MRS, composition described below) [32,35], and dialysates were collected over 20 min sampling epochs. Extracellular levels of l-glutamate, GABA, serotonin, norepinephrine and dopamine were measured at 8-h after the start of perfusions. After baseline recording, perfusion medium was replaced with MRS containing MK801, muscimol, or perampanel as indicated. Dialysate samples were then injected into the UHPLC (xLC3185PU; Jasco, Tokyo, Japan) apparatus. All samples were taken from freely moving animals.

After microdialysis experiments, brains were removed following cervical dislocation and overdose isoflurane anesthesia. The locations of the dialysis probes were verified in each animal using histological examinations of 200 μm thick brain tissue slices, which were prepared using a Vibratome 1000 (Technical Products International Inc., St. Louis, MO, USA).

### 2.2. Determination of Extracellular Levels of l-glutamate, GABA, Dopamine, Norepinephrine and Serotonin

Concentrations of l-glutamate and GABA were determined using UHPLC (xLC3185PU; Jasco) with fluorescence resonance energy transfer detection (xLC3120FP; Jasco) after dual derivatization with isobutyryl-l-cysteine and *o*-phthalaldehyde. Derivative reagent solutions were prepared by dissolving isobutyryl-l-cysteine (2 mg) or *o*-phthalaldehyde (1 mg) in 0.1 mL aliquots of ethanol, followed by addition of 0.9 mL of sodium borate buffer (0.2 M, pH 9.0) [15,16,32,35]. Automated precolumn derivation was conducted by mixing 5 μL sample, standard, or blank solutions with 5 μL of derivative reagent solution in reaction vials for 5 min before injection (xLC3059AS; Jasco). Derivative samples (5 μL) were injected using an autosampler (xLC3059AS; Jasco). The analytical column (YMC Triart C18, particle 1.8 μm, 50 × 2.1 mm; YMC, Kyoto, Japan) was maintained at 45 °C. The flow rate was set at 500 μL/min, and elution was performed using a linear gradient of mobile phases A (0.05 M acetate buffer, pH 5.0) and B (0.05 M acetate buffer containing 60% acetonitrile, pH 3.5) over 10 min [15,16,32,35]. Excitation and emission wavelengths of the fluorescence detector were set at 280 and 455 nm, respectively.

Concentrations of dopamine, norepinephrine and serotonin were determined using UHPLC (xLC3185PU; Jasco) with electrochemical detection (ECD-300; Eicom) by a graphite carbon electrode set at +450 mV (vs. a Ag/AgCl reference electrode [15,16,32,35]. The analytical column (Triart C18, particle 1.8 μm, 30 × 2.1 mm; YMC) was maintained at 40 °C and the flow rate of the mobile phase was set at 400 μL/min. The mobile phase contained 0.1 M acetate buffer, 1% methanol, and 50 mg/L EDTA-2Na (final pH 6.0) [36]. Where possible, we randomized and blinded sample data. In particular, for determinations of extracellular transmitter levels, the sample order was dictated by the autosampler according to a random number table.

### 2.3. Data Analysis

Where possible, we randomized and blinded sample data. To determine extracellular transmitter levels, the sample order was set on the autosampler according to a random number table. Drug doses and sample sizes were selected according to previous studies [15,16,17,18,19,20,21]. All experiments in this study were designed with equally sized animal groups (*n* = 6) [15,16,17,18,19,20,21] and all values were expressed as mean ± SD. Differences were considered significant when *p* < 0.05 (two-tailed). Regional transmitter concentrations were analyzed using Mauchly’s sphericity test followed by multivariate analysis of variance (MANOVA) using BellCurve for Excel ver. 3.20 (Social Survey Research Information Co., Ltd., Tokyo, Japan). When the data did not violate the assumption of sphericity (*p* > 0.05), the F value of MANOVA was analyzed using sphericity-assumed degrees of freedom. When the assumption of sphericity was violated (*p* < 0.05), F values were analyzed using Chi–Muller’s corrected degrees of freedom by BellCurve for Excel. When F values for drug factors were significant in MANOVA, the data were finally analyzed using Tukey’s post hoc test with BellCurve for Excel.

### 2.4. Chemical Agents

The noncompetitive NMDAR antagonist, MK801 (dizocilpine) and the GABA_A_-R agonist, muscimol, were obtained from Fujifilm-Wako (Osaka, Japan). The AMPAR antagonist, perampanel was obtained from Cosmo Bio (Tokyo, Japan). These compounds were prepared on the day of experiments. These drugs were perfused in MRS containing 145 mM Na^+^, 2.7 mM K^+^, 1.2 mM Ca^2+^, 1.0 mM Mg^2+^, and 154.4 mM Cl^−^, which was adjusted to pH 7.4 using 2 mM phosphate buffer and 1.1 mM Tris buffer [15,16]. Muscimol and MK801 were dissolved in MRS directly. Perampanel was initially dissolved at a concentration of 1 mM in dimethyl sulfoxide.

According to previous reports, in the present study, to study effects of MK801, muscimol and perampanel, each rat was administrated by perfusion with MK801 (1, 5 and 50 μM) [15,16,17,20,21], muscimol (1 μM) [23,24] and perampanel (1 μM) [19,20] into the mPFC, RTN or DRN.

## 3. Results

### 3.1. Effects of NMDAR in the mPFC on the Regional Transmitter Release (Study_1)

Previously, perfusion with 50 μM MK801 into the mPFC (medial prefrontal cortex) increased the regional extracellular levels of serotonin, norepinephrine and dopamine, but decreased that of GABA without affecting l-glutamate level [16,23,24,25]; however, the effects of lower than 50 μM MK801 on the extracellular transmitter levels in the mPFC have not been clarified. To explore the threshold level of local administration (perfusion with) MK801 into the mPFC, Study_1 was designed to determine the effects of perfusion with lower than 50 μM MK801 into the mPFC on the regional extracellular levels of l-glutamate, GABA, serotonin, norepinephrine and serotonin. Additionally, the major mechanisms of 50 μM mPFC MK801-induced monoamine release was considered to be modulated by regional GABAergic disinhibition [16,23,24,25]. Therefore, to clarify the mechanisms of MK801-induced monoamine release, the effects of perfusion with 1 μM muscimol (GABA_A_-R agonist) into the mPFC on the monoamine release induced by threshold concentration of MK801 were determined.

#### 3.1.1. Concentration-Dependent Effects of Local Administration of MK801 into the mPFC on Regional Extracellular Transmitter Levels

**Figure 1:**
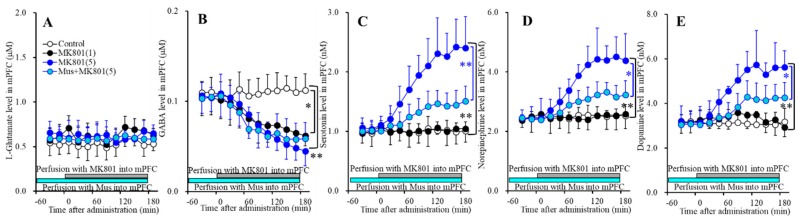
Concentration-dependent effects of local administration of MK801 (0, 1 and 5 μM) into the mPFC on extracellular levels of l-glutamate (**A**), GABA (**B**), serotonin (**C**), norepinephrine (**D**), and dopamine (**E**) in the mPFC (Study_1). Ordinates: mean ± SD (*n* = 6) of extracellular transmitter levels (μM or nM); abscissa: time after MK801 administration (min). Light blue and gray bars indicate perfusion with muscimol (Mus) and MK801 into the mPFC, respectively. * *p* < 0.05, ** *p* < 0.01; relative to control or 5 μM MK801 by multivariate analysis of variance (MANOVA) with Tukey’s post hoc test.

Perfusions with MK801 (1 and 5 μM) into the mPFC increased regional extracellular levels (mPFC MK801-induced release) of serotonin [F_MK801_(2,15) = 23.1 (P<0.05), F_Time_(3.2,47.3) = 41.7 (*p* < 0.05), F_MK801*Time_(6.3,47.3) = 36.9 (*p* < 0.05)] (Figure 1C), norepinephrine [F_MK801_(2,15) = 20.9 (*p* < 0.05), F_Time_(3.6,54.3) = 43.7 (*p* < 0.05), F_MK801*Time_(7.2,54.3) = 36.4 (*p* < 0.05)] (Figure 1D) and dopamine [F_MK801_(2,15) = 18.8 (*p* < 0.05), F_Time_(3.4,51.6) = 32.0 (*p* < 0.05), F_MK801*Time_(6.9,51.6) = 25.9 (*p* < 0.05)] (Figure 1E), but decreased GABA level [F_MK801_(2,15) = 7.4 (*p* < 0.05), F_Time_(5.7,84.9) = 56.2 (*p* < 0.05), F_MK801*Time_(11.3,84.9) = 22.2 (*p* < 0.05)] (Figure 1B) without affecting l-glutamate level in the mPFC (Figure 1A). Extracellular levels of monoamine (serotonin, norepinephrine and dopamine) were increased by 5 μM MK801 but not by 1 μM MK801 (Figure 1C–E); however, extracellular GABA level was decreased by 1 μM and 5 μM MK801 (Figure 1B). Therefore, the threshold concentration of local administration of MK801 into the mPFC on GABA and monoamine are lower than 1 μM and 5 μM, respectively.

#### 3.1.2. Interaction between NMDAR and GABA_A_-R in the mPFC on Regional Extracellular Transmitter Levels

Perfusions with 1 μM muscimol into the mPFC did not affect regional extracellular levels of l-glutamate, GABA, serotonin, norepinephrine, or dopamine (Figure 1). Perfusion with 1 μM muscimol inhibited MK801-induced releases of serotonin [F_MK801_(1,10) = 11.4 (*p* < 0.05), F_Time_(2.8,28.1) = 74.3 (*p* < 0.05), F_MK801*Time_(2.8,28.1) = 17.0 (*p* < 0.05)]) (Figure 1C), norepinephrine [F_MK801_(1,10) = 9.1 (*p* < 0.05), F_Time_(2.7,26.6) = 65.2 (*p* < 0.05), F_MK801*Time_(2.7,26.6) = 12.1 (*p* < 0.05)] (Figure 1D) and dopamine [F_MK801_(1,10) = 7.3 (*p* < 0.05), F_Time_(2.5,25.2) = 47.1(*p* < 0.05), F_MK801*Time_(2.5,25.2) = 5.3(*p* < 0.05)] (Figure 1E) without affecting MK801-induced reduction of GABA release (Figure 1B). These results suggest that MK801 increases monoamine release in the mPFC (threshold level is 5 μM) via regional GABAergic disinhibition (threshold level is 1 μM).

### 3.2. Effects of NMDAR in the RTN on Transmitter Releases in the mPFC and MDTN (Study_2)

Several previous reports demonstrated that the activation of thalamocortical glutamatergic pathway is one of the major responsible glutamatergic pathways of systemic MK801-induced l-glutamate release in the mPFC (medial prefrontal cortex) [15,17,18,20,21]. Our previous studies demonstrated that activation of glutamatergic neurons in the MDTN (mediodorsal thalamic nucleus) increased the l-glutamate release in the mPFC, IsC (insular cortex) and OFC (orbitofrontal cortex) [15,17,18,20,21]. The threshold concentrations of local administration of MK801 into the MDTN on l-glutamate release in the MDTN and mPFC were 50 μM [17,21]. Contrary to the MDTN, the threshold concentrations of local administration of MK801 into the RTN (reticular thalamic nucleus) on l-glutamate release in the MDTN and mPFC were 5 μM and 1 μM, respectively [17,21]. These finding suggest that the MDTN is responsible region for thalamocortical glutamatergic transmission, but a candidate generator region is the RTN. To explore the threshold concentration of local administration of MK801 into the RTN on thalamocortical glutamatergic transmission and its associated other transmitter release, Study_2 was designed to determine the effects of MK801 (1, 5 and 50 μM) into the RTN on the releases of l-glutamate, GABA, serotonin, norepinephrine, and dopamine in the mPFC and MDTN.

#### 3.2.1. Concentration-Dependent Effects of Local Administration of MK801 into the RTN on Extracellular Transmitter Levels in the mPFC

**Figure 2:**
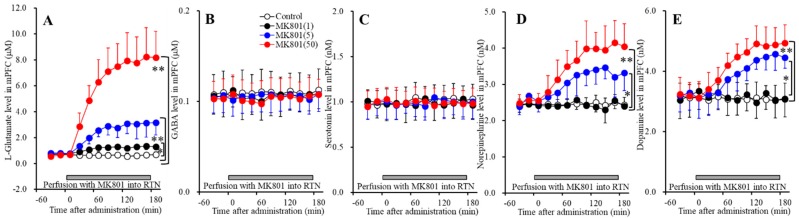
Concentration-dependent effects of local administration of MK801 (1, 5, and 50 μM) into the RTN on extracellular levels of l-glutamate (**A**), GABA (**B**), serotonin (**C**), norepinephrine (**D**), and dopamine (**E**) in the mPFC (Study_2). Ordinates: mean ± SD (*n* = 6) of extracellular transmitter levels (μM or nM); abscissa: Time after MK801 administration (min). Gray bars indicate perfusion with MK801 into the RTN. * *p* < 0.05, ** *p* < 0.01; relative to control by MANOVA with Tukey’s post hoc test.

Perfusions with MK801 (1, 5 and 50 μM) into the RTN increased extracellular levels of l-glutamate [F_MK801_(3,20) = 55.0 (*p* < 0.05), F_Time_(2.3,46.8) = 99.6 (*p* < 0.05), F_MK801*Time_(7.0,46.8) = 43.3 (*p* < 0.05)] (Figure 2A), norepinephrine [F_MK801_(3,20) = 16.4 (*p* < 0.05), F_Time_(5.9,118.2) = 46.7 (*p* < 0.05), F_MK801*Time_ (17.7,118.2) = 18.5 (*p* < 0.05)] (Figure 2D), and dopamine [F_MK801_(3,20) = 9.0 (*p* < 0.05), F_Time_(5.7,114.7) = 49.2 (*p* < 0.05), F_MK801*Time_(17.2,114.7) = 21.3 (*p* <0.05)] (Figure 2E) without affecting those of GABA or serotonin in the mPFC (Figure 2B,C). Extracellular levels of catecholamine (norepinephrine and dopamine) were increased by 5 μM MK801 but not by 1 μM MK801 (Figure 2D,E); however, extracellular l-glutamate level was increased by both 1 μM and 5 μM MK801 (Figure 2A). Therefore, the threshold concentration of local administration of MK801 into the RTN on releases of l-glutamate and catecholamine in the mPFC are 1 μM and 5 μM, respectively.

#### 3.2.2. Concentration-Dependent Effects of Local Administration of MK801 into the RTN on Extracellular Transmitter Levels in the MDTN

**Figure 3:**
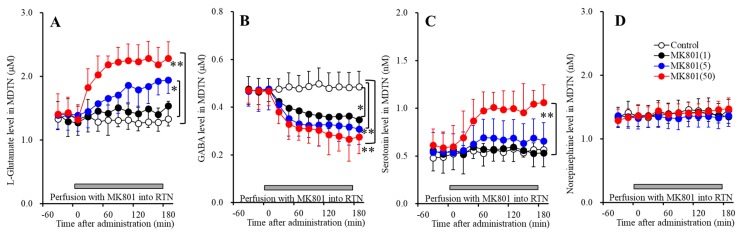
Concentration-dependent effects of local administration of MK801 (1, 5, and 50 μM) into the RTN on extracellular levels of l-glutamate (**A**), GABA (**B**), serotonin (**C**), and norepinephrine (**D**) in the MDTN (Study_2). Ordinates: mean ± SD (*n* = 6) of extracellular transmitter levels (μM or nM); abscissa: time after MK801 administration (min). Gray bars indicate perfusion with MK801 into the RTN. * *p* < 0.05, * *p* < 0.01; relative to control by MANOVA with Tukey’s post hoc test.

Perfusions with MK801 (1, 5 and 50 μM) into the RTN increased extracellular levels of l-glutamate [F_MK801_(3,20) = 11.4 (*p* < 0.05), F_Time_(9,180) = 50.5 (*p* < 0.05), F_MK801*Time_(27,180) = 17.8 (*p* < 0.05)] (Figure 3A) and serotonin [F_MK801_(3,20) = 7.2 (*p* < 0.05), F_Time_(4.9,98.0) = 21.3 (*p* < 0.05), F_MK801*Time_(14.7,98.0) = 7.5 (*p* < 0.05)] (Figure 3C), and decreased GABA [F_MK801_(3,20) = 9.0 (*p* < 0.05), F_Time_(6.4,128.6) = 73.7 (*p* < 0.05), F_MK801*Time_(19.3,128.6) = 12.6 (*p* < 0.05)] (Figure 3B) without affecting those of norepinephrine in the MDTN (Figure 3D). The extracellular dopamine level in the MDTN could not be detected.

Extracellular serotonin level was increased by 50 μM MK801 but not by 1 μM or 5 μM MK801 (Figure 3C). Extracellular GABA level was decreased by 1 μM MK801 (Figure 3B). Extracellular l-glutamate level was increased by 5 μM MK801, but not by 1 μM MK801 (Figure 3A). Therefore, the threshold concentrations of local administration of MK801 into the RTN on releases of l-glutamate, GABA and serotonin in the MDTN are 5 μM, 1 μM, and 50 μM, respectively.

### 3.3. Interaction between NMDAR, GABA_A_-R and AMPAR in the mPFC and MDTN on Extracellular Transmitter Levels in the mPFC (Study_3)

The specific noradrenergic, dopaminergic and serotonergic terminals from respective LC (locus coeruleus), VTA (ventral tegmental area) and DRN (dorsal raphe nucleus) project to deeper layers of frontal cortex [15,18,21,23,24,25,26,33,37]. These specific monoaminergic terminals receive directly inhibitory cortical GABAergic regulation, but not excitatory glutamatergic regulation [23,24]. Contrary, co-releasing terminals of norepinephrine and dopamine from LC project to superficial layers in frontal cortex, which receives directly excitatory thalamocortical glutamatergic regulation but not inhibitory GABAergic regulation [15,18,21,23,24,25,26,33,37]. The serotonergic terminal in the superficial layers have not been identified [23,24,25]. Based on these previous findings, to explore the regulation mechanisms of thalamocortical glutamatergic transmission and its associated other transmitter release in the MDTN (mediodorsal thalamic nucleus) and mPFC (medial prefrontal cortex), Study_3 was designed to determine the effects of local administration of 1 μM muscimol (GABA_A_-R agonist) and perampanel (AMPAR antagonist) into the MDTN and mPFC on transmitter releases in the mPFC and MDTN induced by 50 μM MK801 perfusion into the RTN (reticular thalamic nucleus).

#### 3.3.1. Effects of GABA_A_-R and AMPAR in the mPFC and MDTN on RTN MK801-Induced Releases in the mPFC

**Figure 4:**
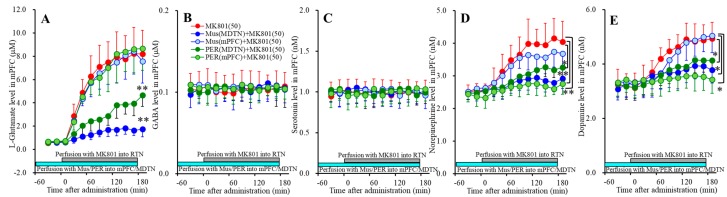
Effects of local administration of muscimol and perampanel into the medial prefrontal cortex (mPFC) and mediodorsal thalamic nucleus (MDTN) on 50 μM RTN MK801-induced releases of l-glutamate (**A**), GABA (**B**), serotonin (**C**), norepinephrine (**D**), and dopamine (**E**) in the mPFC (Study_3). Ordinates: mean ± SD (*n* = 6) of extracellular transmitter levels (μM or nM); abscissa: time after MK801 administration (min). Light blue bars indicate perfusion with 1 μM muscimol (Mus) or perampanel (PER) into the mPFC or MDTN. Gray bars indicate the perfusion with 50 μM MK801 into the RTN. * *p* < 0.05, ** *p* < 0.01; relative to control (50 μM MK801) by MANOVA with Tukey’s post hoc test.

Perfusion with 1 μM muscimol (GABA_A_-R agonist) [F_Mus_(1,10) = 52.4 (*p* <0.05), F_Time_(2.3,22.7) = 82.0 (*p* < 0.05), F_Mus*Time_(2.3,22.7) = 43.8 (*p* < 0.05)] and 1 μM perampanel (AMPAR antagonist) [F_PER_(1,10) = 21.9 (*p* < 0.05), F_Time_(2.3,23.0) = 118.2 (*p* < 0.05), F_PER*Time_(2.3,23.0) = 18.2 (*p* < 0.05)] into MDTN reduced 50 μM RTN MK801-induced l-glutamate release in the mPFC (Figure 4A), whereas neither perfusion with 1 μM muscimol nor perampanel into the mPFC affected 50 μM RTN MK801-induced l-glutamate release in the mPFC (Figure 4A).

Contrary to l-glutamate, 50 μM RTN MK801-induced norepinephrine release in the mPFC was inhibited by perfusion with 1 μM perampanel into the MDTN [F_PER_(1,10) = 7.5 (*p* < 0.05), F_Time_(4.5,44.5) = 65.2 (*p* < 0.05), F_PER*Time_(4.5,44.5) = 8.5 (*p* < 0.05)] and mPFC [F_PER_(1,10) = 16.1 (*p* < 0.05), F_Time_(4.8,47.5) = 40.6 (*p* < 0.05), F_PER*Time_(4.8,47.5) = 19.3 (*p* < 0.05)] (Figure 4D). Perfusion with 1 μM muscimol into the MDTN also inhibited 50 μM RTN MK801-induced norepinephrine release in the mPFC [F_Mus_(1,10) = 11.6 (*p* <0.05), F_Time_(3.9,38.5) = 48.1 (*p* <0.05), F_Mus*Time_(3.9,38.5) = 17.8 (*p* <0.05)], but perfusion with 1 μM muscimol into the mPFC did not affect (Figure 4D). Similar to norepinephrine, 50 μM RTN MK801-induced dopamine release in the mPFC was inhibited by perfusion with 1 μM perampanel into the MDTN [F_PER_(1,10) = 5.0 (*p* < 0.05), F_Time_(7.0,69.7) = 107.2 (*p* < 0.05), F_PER*Time_(7.0,69.7) = 9.8 (*p* < 0.05)] and mPFC [F_PER_(1,10) = 9.2 (*p* < 0.05), F_Time_(7.0,70.1) = 53.6 (*p* < 0.05), F_PER*Time_(7.0,70.1) = 36.7 (*p* < 0.05)] (Figure 4E). Perfusion with 1 μM muscimol into the MDTN inhibited 50 μM RTN MK801-induced dopamine release in the mPFC [F_Mus_(1,10) = 5.3 (*p* < 0.05), F_Time_(6.2,61.7) = 87.2 (*p* < 0.05), F_Mus*Time_(6.2,61.7) = 22.7 (*p* < 0.05)], but perfusion with muscimol into the mPFC did not affect (Figure 4E).

These results suggest that RTN MK801-induced l-glutamate release in the mPFC is generated by the GABAergic disinhibition and relatively activation of AMPAR in the MDTN, but is not modulated by these receptors in the mPFC. Contrary to l-glutamate, RTN MK801-induced catecholamine release in the mPFC is regulated by the GABAergic disinhibition in the MDTN, and activation of AMPAR in the MDTN and mPFC, but is not modulated by GABA_A_-R in the mPFC. Therefore, RTN MK801-induced catecholamine release in the mPFC is activated by AMPAR in the mPFC via activation of thalamocortical glutamatergic transmission.

#### 3.3.2. Effects of GABA_A_-R and AMPAR in the MDTN on RTN MK801-Induced Releases in the MDTN

**Figure 5:**
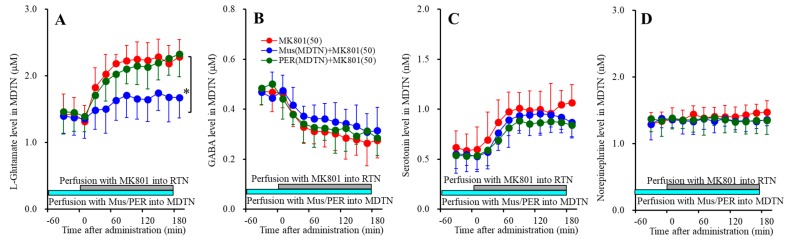
Effects of local administration of muscimol and perampanel into the MDTN on 50 μM RTN MK801-induced releases of l-glutamate (**A**), GABA (**B**), serotonin (**C**), and norepinephrine (**D**) in the MDTN (Study_3). Ordinates: mean ± SD (*n* = 6) of extracellular transmitter levels (μM or nM); abscissa: time after MK801 administration (min). Light blue bars indicate perfusion with 1 μM muscimol (Mus) or perampanel (PER) into the MDTN. Gray bars indicate the perfusion with 50 μM MK801 into the RTN. * *p* < 0.05; relative to control (50 μM MK801) by MANOVA with Tukey’s post hoc test.

Perfusion with 1 μM muscimol (GABA_A_-R agonist) into MDTN reduced 50 μM RTN MK801-induced l-glutamate release in the MDTN[F_PER_(1,10) = 9.3 (*p* < 0.05), F_Time_(5.6,56.3) = 46.9 (*p* < 0.05), F_PER*Time_(5.6,56.3) = 9.9 (*p* < 0.05)], whereas perfusion with 1 μM perampanel (AMPAR antagonist) into the MDTN did not affect (Figure 5A). Contrary to l-glutamate, neither 50 μM RTN MK801-induced serotonin release nor reduction of GABA in the MDTN were affected by perfusion with 1 μM muscimol and perampanel into the MDTN (Figure 5B,C).

These results suggest that RTN MK801-induced l-glutamate release in the MDTN is generated by the GABAergic disinhibition in the MDTN, but is not modulated by AMPAR in the MDTN. Contrary to l-glutamate, RTN MK801-induced serotonin release and reduction of GABA release in the MDTN are not affected by GABA_A_-R or AMPAR in the MDTN.

### 3.4. Effects of NMDAR in the DRN on Transmitter Releases in the DRN, mPFC and MDTN (Study_4)

The results of stdies_1~3 indicated the serotonergic transmission in the mPFC (medial prefrontal cortex) was regulated by independent system compared with other catecholamine, since serotonin release in the mPFC is not affected by thalamocortical glutamatergic transmission, but conversely mesothalamic serotonergic transmission possibly affects thalamocortical glutamatergic pathway in the MDTN (mediodorsal thalamic nucleus) [16]. To clarify the effects of serotonergic transmission on mesocortical serotonergic transmission (DRN-mPFC) and mesothalamic serotonergic transmission (DRN-MDTN), study_4 was designed to determine the effects of local administration of MK801 into the DRN (dorsal raphe nucleus) on serotonin release in the mPFC, DRN and MDTN.

#### 3.4.1. Concentration-Dependent Effects of Local Administration of MK801 into the DRN on Extracellular Transmitter Levels in the mPFC

**Figure 6:**
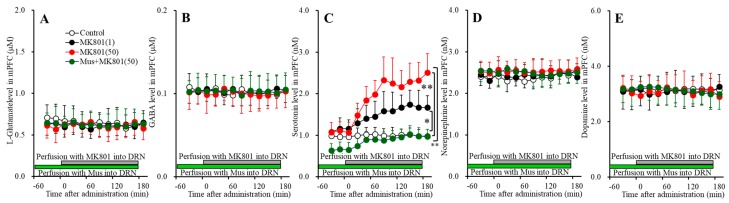
Concentration-dependent effects of local administration of MK801 (1 and 50 μM) into the DRN on extracellular levels of l-glutamate (**A**), GABA (**B**), serotonin (**C**), norepinephrine (**D**), and dopamine (**E**) in the mPFC (Study_4). Ordinates: mean ± SD (*n* = 6) of extracellular transmitter levels (μM or nM); abscissa: time after MK801 administration (min). Green and gray bars indicate perfusion with muscimol and MK801 into the DRN, respectively. * *p* < 0.05, ** *p* < 0.01; relative to control or 50 μM MK801 by MANOVA with Tukey’s post hoc test.

Perfusions with MK801 (1 and 50 μM) into the DRN concentration-dependently increased extracellular serotonin level in the mPFC [F_MK801_(2,15) = 16.8 (*p* < 0.05), F_Time_(4.9,72.8) = 42.9 (*p* < 0.05), F_MK801*Time_(9.7,72.8) = 16.1 (*p* < 0.05)] (Figure 6C) without affecting those of l-glutamate, GABA, norepinephrine or dopamine (Figure 6A,B,D,E). Extracellular serotonin level was increased by 1 μM and 50 μM MK801 (Figure 6C). Therefore, the threshold concentration of local administration of MK801 into the DRN on serotonin release in the mPFC is lower than 1 μM.

Perfusion with 1 μM muscimol into the DRN decreased the basal and DRN 50 μM MK801-induced serotonin release in the mPFC [F_MK801_(1,10) = 41.9 (*p* < 0.05), F_Time_(3.4,34.1) = 63.8 (*p* < 0.05), F_MK801*Time_(3.4,34.1) = 24.0 (*p* < 0.05)] (Figure 6C) without affecting those of l-glutamate, GABA, norepinephrine or dopamine (Figure 6A,B,D,E). Therefore, DRN MK801-induced serotonin release in the mPFC is probably induced by GABAergic disinhibition in the DRN.

#### 3.4.2. Concentration-Dependent Effects of Local Administration of MK801 into the DRN on Extracellular Transmitter Levels in the MDTN

**Figure 7:**
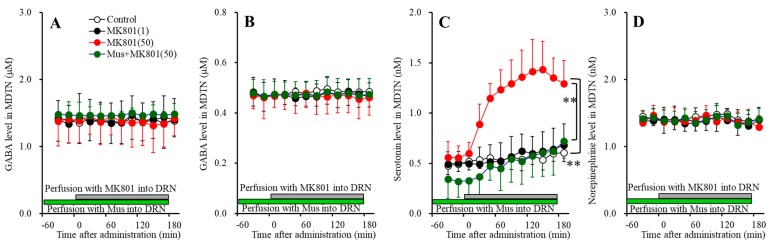
Concentration-dependent effects of local administration of MK801 (1 and 50 μM) into the DRN on extracellular levels of l-glutamate (**A**), GABA (**B**), serotonin (**C**) and norepinephrine (**D**) in the MDTN (Study_4). Ordinates: mean ± SD (*n* = 6) of extracellular transmitter levels (μM or nM); abscissa: time after MK801 administration (min). Green and gray bars indicate Perfusion with muscimol and MK801 into the DRN, respectively. * *p* < 0.05, ** *p* < 0.01; relative to control or 50 μM MK801 by MANOVA with Tukey’s post hoc test.

Perfusions with MK801 (1 and 50 μM) into the DRN concentration-dependently increased extracellular serotonin level in the MDTN [F_MK801_(2,15) = 11.6 (*p* < 0.05), F_Time_(6.9,103.6) = 26.0 (*p* < 0.05), F_MK801*Time_(13.8,103.6) = 6.3 (*p* < 0.05)] (Figure 7C) without affecting those of l-glutamate, GABA, norepinephrine or dopamine (Figure 7A,B,D). Extracellular serotonin level was increased by 50 μM MK801, but not by 1 μM MK801 (Figure 7C). Our previous study demonstrated that local administration of 5 μM MK801 into the DRN increased serotonin release in the MDTN [16]. Therefore, taken together with our previous demonstration, the threshold concentration of local administration of MK801 into the DRN on serotonin release in the DRN is 5 μM.

Perfusion with 1 μM muscimol into the DRN decreased the basal and DRN 50 μM MK801-induced serotonin release in the MDTN [F_MK801_(1,10) = 21.5 (*p* < 0.05), F_Time_(5.2,51.9) = 36.3 (*p* < 0.05), F_MK801*Time_(5.2,51.9) = 6.1 (*p* < 0.05)] (Figure 7C). Therefore, the serotonin release in the MDTN is probably generated by the activation of serotonergic neurons in the DRN, similar to serotonin release in the mPFC.

#### 3.4.3. Concentration-Dependent Effects of Local Administration of MK801 into the DRN on Extracellular Transmitter Levels in the DRN

**Figure 8:**
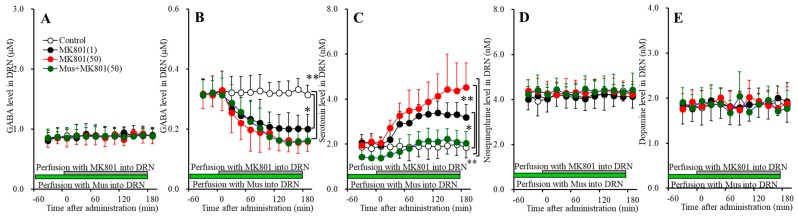
Concentration-dependent effects of local administration of MK801 (1 and 50 μM) into the DRN on extracellular levels of l-glutamate (**A**), GABA (**B**), serotonin (**C**), norepinephrine (**D**) and dopamine (**E**) in the DRN (Study_4). Ordinates: mean ± SD (*n* = 6) of extracellular transmitter levels (μM or nM); abscissa: time after MK801 administration (min). Green and gray bars indicate perfusion with muscimol and MK801 into the DRN, respectively. * *p* < 0.05, ** *p* < 0.01; relative to control or 50 μM MK801 by MANOVA with Tukey’s post hoc test.

Perfusions with MK801 (1 and 50 μM) into the DRN concentration-dependently increased and decreased extracellular levels of serotonin [F_MK801_(2,15) = 13.6 (*p* < 0.05), F_Time_(5.1,76.3) = 53.3 (*p* < 0.05), F_MK801*Time_(3.4,34.1) = 15.8 (*p* < 0.05)] (Figure 8C) and GABA [F_MK801_(2,15) = 9.9 (*p* < 0.05), F_Time_(4.8,72.0) = 42.9 (*p* < 0.05), F_MK801*Time_(9.6,72.0) = 12.1 (*p* < 0.05)] (Figure 8B) in the DRN, respectively. Contrary to them, perfusions with MK801 (1 and 50 μM) into the DRN did not affect extracellular levels of l-glutamate, norepinephrine or dopamine in the DRN (Figure 8A,D,E). Extracellular levels of GABA and serotonin were decreased and increased by 1 μM MK801, respectively (Figure 8B,C). Therefore, the threshold concentrations of local administration of MK801 into the DRN on releases of GABA and serotonin in the DRN are 1 μM.

Perfusion with 1 μM muscimol into the DRN decreased the basal and DRN 50 μM MK801-induced serotonin release in the DRN [F_MK801_(1,10) = 22.0 (*p* < 0.05), F_Time_(3.7,37.4) = 42.0 (*p* < 0.05), F_MK801*Time_(3.7,37.4) = 6.1 (*p* < 0.05)] (Figure 8C), whereas perfusion with 1 μM muscimol into the DRN did not affect basal or 50 μM MK801-induced GABA reduction in the DRN (Figure 8B). These results indicate that DRN MK801-induced serotonin release in the mPFC, MDTN and DRN were generated by GABAergic disinhibition within the DRN.

## 4. Discussion

The threshold concentration of local administration (perfusion) of MK801 into the mPFC, MDTN, RTN, and DRN on several transmission systems demonstrated by this study and previous reports [15,16,17,20] are summarized in the Table 1.

**Figure 9:**
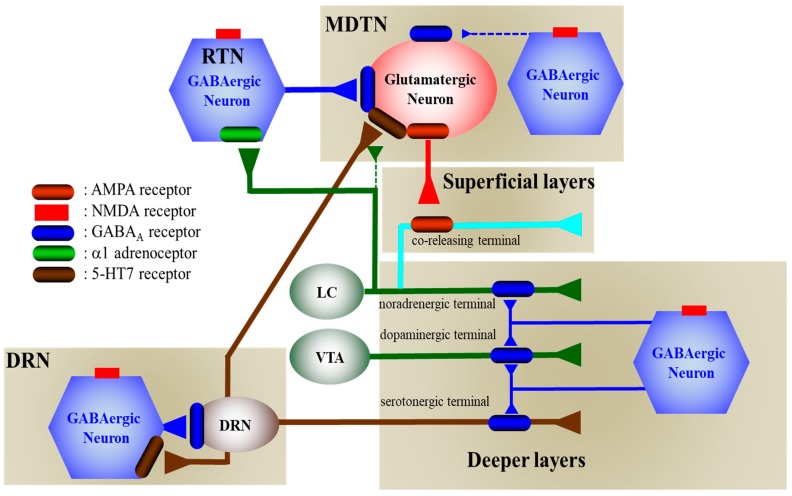
Proposed hypothesis for the extended neural circuitry involved in thalamocortical (RTN–MDTN–mPFC) glutamatergic, mesothalamic (DRN-MDTN), and mesocortical (DRN-mPFC) serotonergic connectivities and their regulation systems. Glutamatergic neurons in the MDTN, which receive GABAergic terminal from RTN and other MDTN region, serotonergic terminal from DRN, noradrenergic terminal from LC and dopaminergic terminal from VTA projects to the mPFC. Inhibitory GABAergic regulation on MDTN glutamatergic neuron from the RTN prevails over that from the MDTN. MK801 inhibits tonically active NMDAR (red squares) on GABAergic neurons in the RTN and MDTN. Inhibition of NMDAR on GABAergic neurons leads to disinhibition of MDTN glutamatergic neurons. The GABAergic disinhibition activates MDTN glutamatergic neuronal activity resulting in an increase in glutamate release in the mPFC. The catecholaminergic terminals but not serotonergic terminals receive excitatory glutamatergic projection which activates AMPAR on the catecholamine co-releasing terminal in the superficial layers of mPFC. The selective serotonergic, noradrenergic and dopaminergic terminals in the deeper layers of mPFC receive inhibitory GABAergic projection from other cortexes. Serotonergic neurons (brown circle) in the DRN, receive regional GABAergic inhibition, project to the mPFC and MDTN. MK801 inhibits also tonically active NMDAR on GABAergic neurons in the DRN. Inhibition of NMDAR on GABAergic neurons leads to disinhibition of DRN serotonergic neurons. The GABAergic disinhibition activates DRN serotonergic neuronal activity resulting in an increase in serotonin releases in the mPFC and MDTN.

The present study also demonstrated the presence of several regulatory systems in the thalamocortical (RTN-MDTN-mPFC) glutamatergic, mesothalamic (DRN-MDTN), and mesocortical (DRN-mPFC) serotonergic pathways controlling releases of l-glutamate, GABA, serotonin, norepinephrine and dopamine in the mPFC, MDTN and DRN. According to the results in this study and published neural circuits [15,16,18,20,21,24,30,38,39,40], our proposed hypothesis regarding the neural networks associated with NMDAR was indicated in Figure 9.

### 4.1. Catecholaminergic Transmission Regulation System Associated with NMDAR

Local administration of MK801 into the mPFC increased and decreased regional respective monoamine and GABA releases without affecting l-glutamate release [15,20,21,24,30]. These mPFC MK801-induced monoamine release was inhibited by activation of GABA_A_-R in the mPFC (perfusion with 1 μM muscimol into the mPFC). Therefore, inhibition of NMDAR in the mPFC increases monoamine release induced by presynaptic GABAergic disinhibition in the mPFC. Indeed, the threshold concentrations of local administration of MK801 into the mPFC on GABA release (1 μM) is more sensitive compared with that of monoamine (5 μM) (Table 1).

Contrary to intra mPFC regulation system, local administration of MK801 into the RTN increased releases of l-glutamate, norepinephrine and dopamine in the mPFC without affecting those of GABA or serotonin. The threshold concentration of local administration of MK801 into the RTN on releases of l-glutamate and catecholamine (norepinephrine and dopamine) in the mPFC were 1 μM and 5 μM, respectively. The RTN MK801-induced l-glutamate release in the mPFC was inhibited by the activation of GABA_A_-R and inhibition of AMPAR in the MDTN, but was not affected by the activation of GABA_A_-R or inhibition of AMPAR in the mPFC. These results suggest that an activation of glutamatergic neuronal activity induced by GABAergic disinhibition in the MDTN contributes to RTN MK801-induced l-glutamate release in the mPFC.

Various thalamic nuclei, which receive GABAergic inhibition from RTN [39], project excitatory glutamatergic terminals to superficial layers of the mPFC [38,41,42]. Glutamatergic neurons in the MDTN also receive GABAergic inhibition of intra MDTN GABA interneurons [17,20,21], whereas the inhibitory regulation from the RTN on glutamatergic neurons in the MDTN is predominant compared with that from GABAergic interneurons in the MDTN [15,20]. Indeed, the threshold concentration of local administration of MK801 into the MDTN and RTN on GABA release in the MDTN were 50 μM and 1 μM, respectively [15,17,20,21]. Therefore, one of the major responsible pathways of MK801-induced l-glutamate release in the mPFC is thalamocortical (from MDTN to mPFC) glutamatergic pathway, but generating mechanisms is GABAergic disinhibition from RTN to MDTN through NMDAR inhibition in the RTN.

Contrary to l-glutamate release, the RTN MK801-induced catecholamine release in the mPFC was inhibited by the activation of GABA_A_-R in the MDTN, inhibition of AMPAR in the MDTN and mPFC, but was not affected by the activation of GABA_A_-R in the mPFC. Electrophysiological study demonstrated that electrical stimulation of the MDTN increased the releases of glutamate and catecholamine in the mPFC without affecting those of serotonin [23]. Other line studies demonstrated that the afferents from LC compromise two types: the projects to the superficial layer of the mPFC of co-releasing norepinephrine with dopamine, while the other projects to the deep layer of the mPFC as the selective norepinephrine-releasing terminal [23,24,40]. The co-releasing terminals norepinephrine with dopamine from the LC receive excitatory thalamocortical glutamatergic projection which activates AMPAR on the catecholamine co-releasing terminals [23,24]. Taken together with these previous findings, the present study suggests that the hyperactivated glutamatergic transmission in the thalamocortical pathway (from MDTN to mPFC) enhances catecholamine release in the mPFC via activation of AMPAR in the mPFC.

### 4.2. Serotonergic Transmission Regulation System Associated with NMDAR

This study indicates the several specific regulation systems of serotonergic transmission in the mPFC and MDTN which are independent upon catecholamine release regulation system. The first, intra mPFC regulation of serotonergic transmission associated with NMDAR is resembling to the regulation systems of catecholaminergic transmission, since the selective noradrenergic, dopaminergic and serotonergic terminals in the deeper layers of mPFC receive GABAergic inhibition. However, the serotonergic terminal in the mPFC from DRN does not contact with thalamocortical glutamatergic afferents, whereas mesothalamic serotonergic transmission activates thalamocortical glutamatergic transmission, since an activation of serotonergic neuronal activities in the DRN increases serotonin release in the MDTN. Recent multiprobe microdialysis studies demonstrated that an activation of serotonergic neuronal activity enhances MDTN glutamatergic neurons through activation of excitatory 5-HT7 receptor in the MDTN [15,16]. In the present study, the norepinephrine release in the MDTN could be detected, but that of dopamine could not be detected. The present study has not determined the functionally impact of norepinephrine release in the MDTN on thalamocortical glutamatergic transmission, whereas our previous study suggest that the noradrenergic transmission from LC probably attenuates thalamocortical glutamatergic transmission via activates α1 adrenoceptor on the RTN GABAergic neurons, but norepinephrine release in the MDTN does not affect thalamocortical glutamatergic transmission [18]. Therefore, the regulatory effects of norepinephrine and serotonin in the thalamus are independent, since thalamocortical glutamatergic transmission is inhibited and enhanced by activation of respective α1 adrenoceptor on the GABAergic neuron in the RTN and 5-HT7 receptor on the glutamatergic neurons in the MDTN [15,16,18].

In the DRN, serotonin release is regulated by inhibitory GABA_A_-R predominantly, as indicated by decreased regional serotonin release following local administration of muscimol (GABA_A_-R agonist) into the DRN; however, GABA release is regulated by excitatory 5-HT7 receptor, reflecting decreased regional GABA release following local perfusions with SB269970 (5-HT7R antagonist) into the DRN [16]. Previous electrophysiological studies show that inhibitory 5-HT1AR and excitatory 5-HT2A and 5-HT7R response in serotonergic neurons were more than 90% and lower than 20% in the DRN, respectively [43]. In contrast with serotonergic neurons, inhibitory and excitatory 5-HT responses in GABAergic neurons were 15% and 80% in the DRN, respectively, likely relating to 5-HT7R excitatory responses [43]. Taken with these reports, GABA_A_-R and 5-HT7R predominantly inhibit serotonergic neurons and enhance GABAergic neurons, respectively. In the present study, local administration of MK801 into the DRN, decreased and increased releases of GABA and serotonin in the DRN, respectively. The opposite effects of MK801 between releases of GABA and serotonin were inhibited by local administration of muscimol in the DRN. Therefore, NMDAR in the DRN regulates predominantly GABAergic neurons rather than serotonergic neuronal activity in the DRN.

### 4.3. Pharmacological Discrimination of Effects of MK801 on Thalamocortical Glutamatergic Associated Catecholamine Release and Mesocortical Serotonergic Transmission

The present study demonstrated that inhibition of NMDAR directly inhibited GABAergic transmission, but enhanced indirectly glutamatergic and monoaminergic transmissions induced by GABAergic disinhibition. In other words, during resting stage, MK801 selectively inhibits NMDAR on GABAergic neurons, but cannot affect NMDAR on the glutamatergic or monoaminergic neurons. In generally, GABAergic interneuron is more sensitive to NMDAR antagonist compared with other types of neurons, since the resting membrane potential of GABAergic interneurons are more positive (−50 ~−60 mV) rather than those of monoaminergic and glutamatergic neurons [43,44]. NMDAR is voltage-sensitive ligand gated cation channel, since the extracellular magnesium and zinc ions bind to specific sites of NMDAR and blocks cation channel pore during resting stage. However, depolarization (higher than −20 mV) repels magnesium and zinc ions from the channel pore, resulting in voltage-dependently inflow of sodium and calcium ions, and outflow potassium ions [45]. Taken together with these findings, NMDAR on GABAergic neurons can voltage-dependently be activated by small amount of depolarization compared with NMDAR on other glutamatergic and monoaminergic neurons.

In this study, the threshold concentrations of local administrations of MK801 into the mPFC, RTN and DRN on regional GABAergic transmission were almost equal to be 1 μM. Contrary to GABA, the threshold concentrations of local administration of MK801 into the mPFC and RTN on catecholamine release in the mPFC were 5 μM, whereas those into mPFC and DRN on serotonin release in the mPFC were 5 μM and 1 μM, respectively. Therefore, serotonergic transmission is more sensitive to NMDAR antagonist rather than other catecholaminergic transmissions in the mPFC.

Subanesthetic doses of ketamine produces transient dissociative and psychotomimetic effects that resemble the positive and negative symptoms of schizophrenia [46]; however, numerous placebo-controlled studies have demonstrated that sub-anesthetic-dose ketamine exerts rapid, robust, and relatively sustained antidepressant effects in patients with antidepressant-resistant major depressive disorder and bipolar depression [11,47,48,49,50]. Ketamine has also been shown to have distinct and independent anti-suicidal and anti-anhedonic effects in patients with mood disorders [11,12].

Neither clinical mechanisms between psychotomimetic and antidepressant effects of ketamine have remained to be clarified. Local administration of ketamine into the infralimbic mPFC reproduced the antidepressant-like actions of systemic ketamine administration [51]. Local administration of (*R*)-ketamine into the infralimbic mPFC and hippocampus also produced antidepressant-like action [52], whereas local administration of (*R*)-ketamine into the prelimbic mPFC nucleus accumbens could not produce [52]. These findings suggest that infralimbic mPFC, but not prelimbic mPFC, is responsible region of the antidepressant-like action of ketamine and (*R*)-ketamine. Moreover, the binding affinity (Ki values) of (*S*)-ketamine and (*R*)-ketamine to NMDAR are 0.7 and 2.6 μM, respectively [53]. Indeed, the anesthetic effects of (*S*)-ketamine is potent than that of (*R*)-ketamine [54]. The MK801 exhibits rapid antidepressant-like effects, but cannot keep long-lasting its action [55]. Clinical studies have also demonstrated that the antidepressant actions of ketamine against patients with major depression are more potent than those of other NMDAR antagonists, [56,57]. These clinical and preclinical demonstrations suggest that NMDAR inhibition, at least partially, contributes to rapid-acting antidepressant and anti-suicidal actions. Therefore, the superior clinical effects of ketamine against antidepressant-resistant depression is mediated multimodal mechanisms, but NMDAR antagonism plays important roles in the rapid-acting and anti-suicidal action of ketamine. Exactly, a recent preclinical study reported that inhibition of NMDAR-dependent bursting activity in the lateral habenula is probably associated with rapid-acting antidepressant-like effects of ketamine [58].

The thalamocortical glutamatergic transmission plays important roles in the function of neuro-cognition, including learning, memory, and perceptual integration [47,59,60]. The mesocortical serotonergic transmission contributes to improvement of depressive mood [61,62,63], and mesothalamic serotonergic transmission plays important roles in emotional perception via regulation of thalamocortical glutamatergic transmission [15,16,64]. Based on these findings, the present demonstration, the higher sensitivity of mesocortical serotonergic transmission and equivalent sensitivity of mesothalamic serotonergic transmission to MK801 compared with thalamocortical transmission of l-glutamate and secondary catecholamine release in the mPFC, suggests that lower concentration of MK801 affects emotional rather than cognitive disturbances. In the present study, inhibition of AMPAR in the MDTN and mPFC attenuated the MK801-induced glutamatergic and monoaminergic transmissions without affecting GABAergic disinhibition. Several preclinical studies reported the possibility that NMDAR inhibition conversely activates glutamatergic transmission associated with AMPAR through GABAergic disinhibition [58,65]. In spite of suspending clinical trials, the rapid-acting antidepressant-like effects of AMPAR positive allosteric modulator, S47445 have been also demonstrated by preclinical study [66], whereas the other line preclinical study suggested that AMPAR activation may not be necessary for the antidepressant effects of ketamine [67]. The detailed discussion regarding the effects of AMPAR activation and NMDAR inhibition on rapid-acting antidepressant-like action should be needed the results of an FDA approved clinical study using perampanel and ketamine (NCT03367533).

## 5. Conclusions

The present study determined the effects of MK801 on the thalamocortical (RTN-MDTN-mPFC) glutamatergic, mesothalamic (DRN-MDTN), and mesocortical (DRN-mPFC) serotonergic transmissions using multiprobe microdialysis, to clarify the NMDAR associated regulation systems in these three pathways. Inhibition of NMDAR in the RTN activates thalamocortical glutamatergic transmission induced by GABAergic inhibition and secondarily activated AMPAR in the MDTN. The enhanced l-glutamate release in the mPFC activates AMPAR on the regional co-releasing terminals resulting in an increase in releases of norepinephrine and dopamine, but does not affect serotonin release. Inhibition of NMDAR in the DRN enhances serotonin release in the DRN, MDTN and mPFC via GABAergic disinhibition in the DRN. The sensitivities of serotonin release in the mPFC to local administration of MK801 into the DRN is more predominant rather than that of catecholamine release in the mPFC induced by local MK801 administration into the RTN.

## Figures and Tables

**Table 1:** Summary of threshold concentration of MK801.

Administration	Sampling	l-glutamate	GABA	Serotonin	Norepinephrine	Dopamine
mPFC	mPFC (Figure 1)	>50	1 (↓)	5 (↑)	5 (↑)	5 (↑)
MDTN	MDTN	50 (↑) [20]	50 (↓) [17,20]			
	mPFC/IsC	50 (↑) [15]	>50 [17]		50 (↑) [15]	50 (↑) [15]
RTN	MDTN (Figure 3)	5 (↑) [15]	1 (↓) [20]	50 (↑)	>50	
	mPFC/IsC (Figure 2)	1 (↑) [20]	>50	>50	5 (↑) [15]	5 (↑) [15]
DRN	DRN (Figure 8)	>50	1 (↓)	1 (↑)	>50	>50
	MDTN (Figure 7)	>50	>50	5 (↑) [16]	>50	
	mPFC (Figure 6)	>50	>50	1 (↑)	>50	>50

mPFC: medial prefrontal cortex, MDTN: mediodorsal thalamic nucleus, RTN: reticular thalamic nucleus, DRN: dorsal raphe nucleus. Numbers: threshold concentration of MK801 (μM), ↑: increase, ↓: decrease.
